# High-throughput dry transfer and excitonic properties of twisted bilayers based on CVD-grown transition metal dichalcogenides†

**DOI:** 10.1039/d3na00371j

**Published:** 2023-09-04

**Authors:** Hibiki Naito, Yasuyuki Makino, Wenjin Zhang, Tomoya Ogawa, Takahiko Endo, Takumi Sannomiya, Masahiko Kaneda, Kazuki Hashimoto, Hong En Lim, Yusuke Nakanishi, Kenji Watanabe, Takashi Taniguchi, Kazunari Matsuda, Yasumitsu Miyata

**Affiliations:** a Department of Physics, Tokyo Metropolitan University Hachioji 192-0397 Japan ymiyata@tmu.ac.jp wjzhang@tmu.ac.jp; b Department of Materials Science and Engineering, Tokyo Institute of Technology Yokohama 226-8503 Japan; c Department of Chemistry, Saitama University Saitama 338-8570 Japan; d Research Center for Electronic and Optical Materials, NIMS Tsukuba 305-0044 Japan; e Research Center for Materials Nanoarchitectonics, NIMS Tsukuba 305-0044 Japan; f Institute of Advanced Energy, Kyoto University Kyoto 611-0011 Japan

## Abstract

van der Waals (vdW) layered materials have attracted much attention because their physical properties can be controlled by varying the twist angle and layer composition. However, such twisted vdW assemblies are often prepared using mechanically exfoliated monolayer flakes with unintended shapes through a time-consuming search for such materials. Here, we report the rapid and dry fabrication of twisted multilayers using chemical vapor deposition (CVD) grown transition metal chalcogenide (TMDC) monolayers. By improving the adhesion of an acrylic resin stamp to the monolayers, the single crystals of various TMDC monolayers with desired grain size and density on a SiO_2_/Si substrate can be efficiently picked up. The present dry transfer process demonstrates the one-step fabrication of more than 100 twisted bilayers and the sequential stacking of a twisted 10-layer MoS_2_ single crystal. Furthermore, we also fabricated hBN-encapsulated TMDC monolayers and various twisted bilayers including MoSe_2_/MoS_2_, MoSe_2_/WSe_2_, and MoSe_2_/WS_2_. The interlayer interaction and quality of dry-transferred, CVD-grown TMDCs were characterized by using photoluminescence (PL), cathodoluminescence (CL) spectroscopy, and cross-sectional electron microscopy. The prominent PL peaks of interlayer excitons can be observed for MoSe_2_/MoS_2_ and MoSe_2_/WSe_2_ with small twist angles at room temperature. We also found that the optical spectra were locally modulated due to nanosized bubbles, which are formed by the presence of interface carbon impurities. The present findings indicate the widely applicable potential of the present method and enable an efficient search of the emergent optical and electrical properties of TMDC-based vdW heterostructures.

## Abstract 2

A rapid and dry fabrication process was developed to produce twisted multilayers of chemical vapor deposition grown transition metal chalcogenide (TMDC) monolayers.

## Graphical abstract

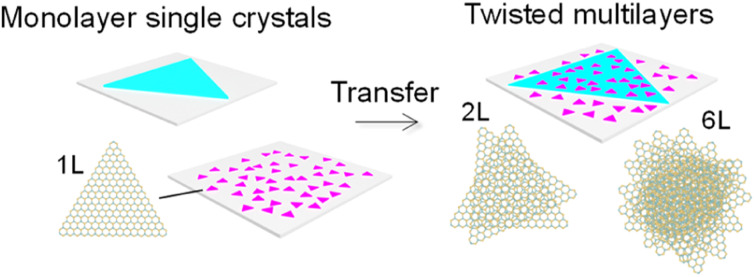

## Introduction

van der Waals (vdW) heterostructures of two-dimensional (2D) materials have recently been studied intensively because of their emergent physical properties and potential applications.^1–7^ In particular, stacked 2D materials with misaligned crystal orientation generate a long-range periodic potential as visualized by a moiré pattern. Because this moiré pattern depends on the twist angle between two layers as well as the constituent materials, many studies have focused on the twist-angle-dependent properties of various 2D materials, such as graphene, hexagonal boron nitride (hBN), and transition metal dichalcogenides (TMDCs). Such twisted vdW systems show various properties including superconductivity,^8^ ferroelectricity,^9,10^ and moiré-related excitonic states.^11–14^ It is noteworthy that this progress has been driven by the development of sophisticated transfer techniques for 2D materials.

Many studies have reported ways of improving the transfer technique of 2D materials with controlled twist angles and various components. The transfer was often conducted by using solution assisted or wet etching processes of substrates.^15–17^ In contrast, dry transfer techniques were also developed to prepare vdW heterostructures with a clean 2D–2D interface.^18–21^ Even though much progress has been made in the dry transfer technique, the search for thin and sufficiently large flakes of 2D materials is still very time-consuming. This is because flakes of 2D materials are usually exfoliated from a bulk layered crystal onto a substrate as a mixture of various layer numbers and shapes. To overcome the limitations posed by this method, current research employs two major approaches. One approach is the use of a robotic search system, which can dramatically reduce the time needed for a person to explore monolayers with sufficient flake size.^22^ The other is the preparation of 2D materials by direct vapor-phase growth rather than by mechanical exfoliation.^23–27^ In particular, recent advances in chemical vapor deposition (CVD) growth enable the preparation of large-area polycrystalline films and single crystals of TMDC monolayers on a wafer-scale substrate.^28–31^ In addition, triangular single crystals of TMDCs enable easy determination of the crystal orientation, allowing easy control of twist angles. Indeed, the use of such CVD-grown polycrystalline TMDC films and single crystals has recently enabled fast, continuous fabrication of vdW heterostructures.^23–27^ For example, Mannix *et al.* demonstrated the fabrication of solid materials consisting of 80 layers of MoS_2_ and twist-controlled 4-layer WS_2_, respectively, by using a robotic assembly with a multi-component polymer stamp.^26^ Despite these great advances, there have been only a few reports on dry transfer and its effect on the optical properties of CVD-grown TMDCs.^26,32^ This is presumably due to the technical difficulties encountered in the growth and dry transfer of CVD-grown TMDC single crystals. One of the major technical difficulties is the efficient peeling of CVD-grown monolayers from growth substrates. In general, CVD-grown monolayers are known to adhere strongly to growth substrates compared to exfoliated flakes. In addition, the adhesion of polymers used for the dry transfer is highly dependent on the process temperatures, which are not optimized for most polymers. Compared with the solution or chemical assisted transfer of CVD grown TMDCs,^33–36^ the dry transfer should keep the intrinsic properties of as grown TMDCs because the solution processes induce physical or chemical property modulations of TMDCs.^37–39^ To expand the versatility of this growth-assisted approach, it is highly desirable to demonstrate the dry transfer and investigate its effects on physical properties.

In this study, we report the rapid and dry fabrication of twisted multilayers of CVD-grown TMDCs using a simple acrylic resin stamp. We introduced the melting and solidification process of an acrylic resin stamp in contact with the sample to improve the adhesion of the stamp. This improvement allows us to efficiently pick up the single crystals of various TMDC monolayers with desired grain size and density from the SiO_2_ surface, and to perform high-throughput and continuous dry transfer. The present process demonstrates the one-step fabrication of more than 100 twisted bilayers, and the creation of 10 layers of MoS_2_ single crystals with different crystal orientations. Furthermore, the hBN encapsulated monolayers and heterobilayers (MoSe_2_/MoS_2_, MoSe_2_/WSe_2_, and MoSe_2_/WS_2_) were created and their twist angle dependent properties were characterized by photoluminescence (PL). We have also investigated the local optical properties of monolayer MoSe_2_ by cathodoluminescence (CL) spectroscopy. The bubbles were observed by cross sectional analysis.

## Results and discussion

**Fig. 1:**
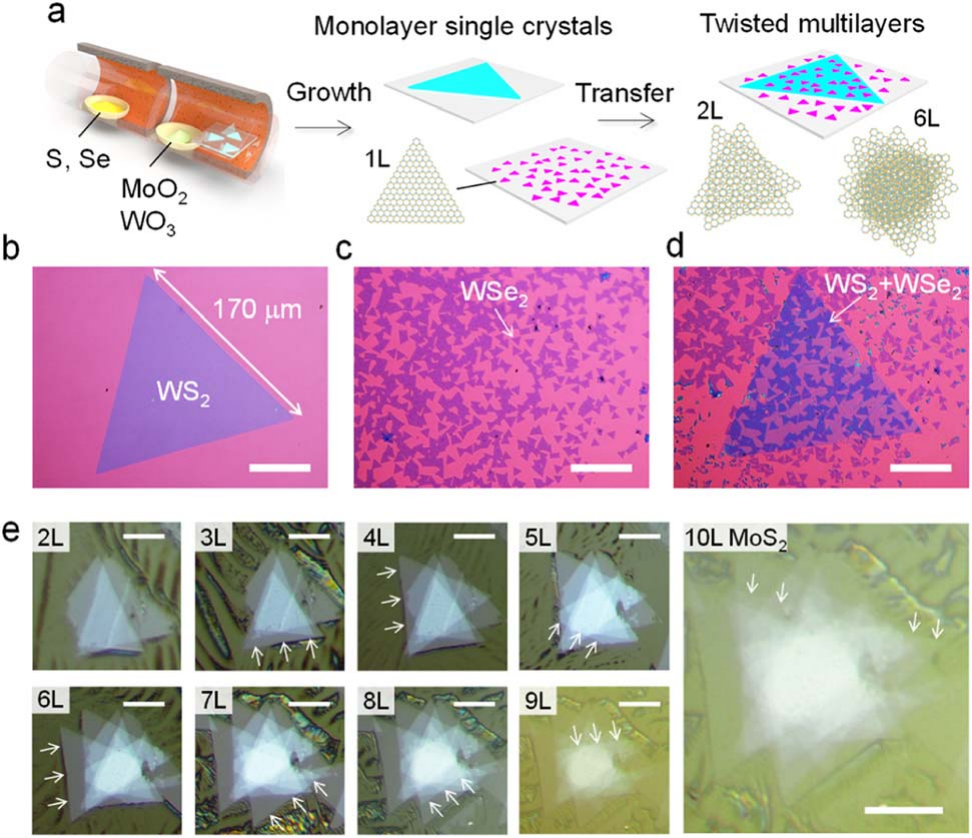
Twisted multilayers fabricated from CVD-grown TMDC monolayers. (a) Schematic of CVD growth of monolayer TMDC single crystals and assemblies into vdW twisted multilayers. Optical images of CVD-grown (b) WS_2_ and (c) WSe_2_ monolayer grains, and (d) stacked bilayers of WS_2_ and WSe_2_. The scale bars are 50 μm. (e) Optical images of 10-layer twisted MoS_2_ in each fabrication step. The scale bars are 10 μm. Arrows indicate newly stacked monolayer MoS_2_ single crystals in each fabrication step.

First, we demonstrate a high-throughput and continuous dry transfer method for creating vdW heterostructures based on a large number of monolayer TMDC single crystals. A major advantage of the CVD process is the controllability of the size and density of single-crystal grains. The small size of the grains ensures the simultaneous transfer of a large number of samples, as shown in Fig. 1a. Fig. 1b and c show the optical images of a large-area WS_2_ single crystal with a size of 170 μm (Fig. 1b) and high-density, small-area WSe_2_ single crystals with a size of around 10 μm (Fig. 1c). Regarding the edge structure, our previous studies have revealed that metal-terminated zigzag edges are formed along the triangular grain edges under the present growth conditions.^40,41^ It should be noted that the exact edge configurations including the edge reconstruction and the step edge cannot be identified simply from the grain shape alone.^42–44^ These individual crystals are oriented in random directions owing to the non-epitaxial growth on the amorphous SiO_2_ surface. First, the large WS_2_ crystal was lifted with a stamp. This large WS_2_ crystal was then used to lift smaller WSe_2_ crystals. Finally, WSe_2_/WS_2_ bilayers were transferred onto a new SiO_2_/Si substrate. This single stacking process yields more than 100 twisted bilayers of WS_2_ and WSe_2_ with different crystal orientations (Fig. 1d). The process was completed in about 1–2 hours. The present dry transfer process enables the continuous stacking of a twisted 10-layer MoS_2_ single crystal (Fig. 1e). The fabrication of such a large number of vdW heterostructures could be useful for exploring the physical properties depending on the twist angle and layer composition.

**Fig. 2:**
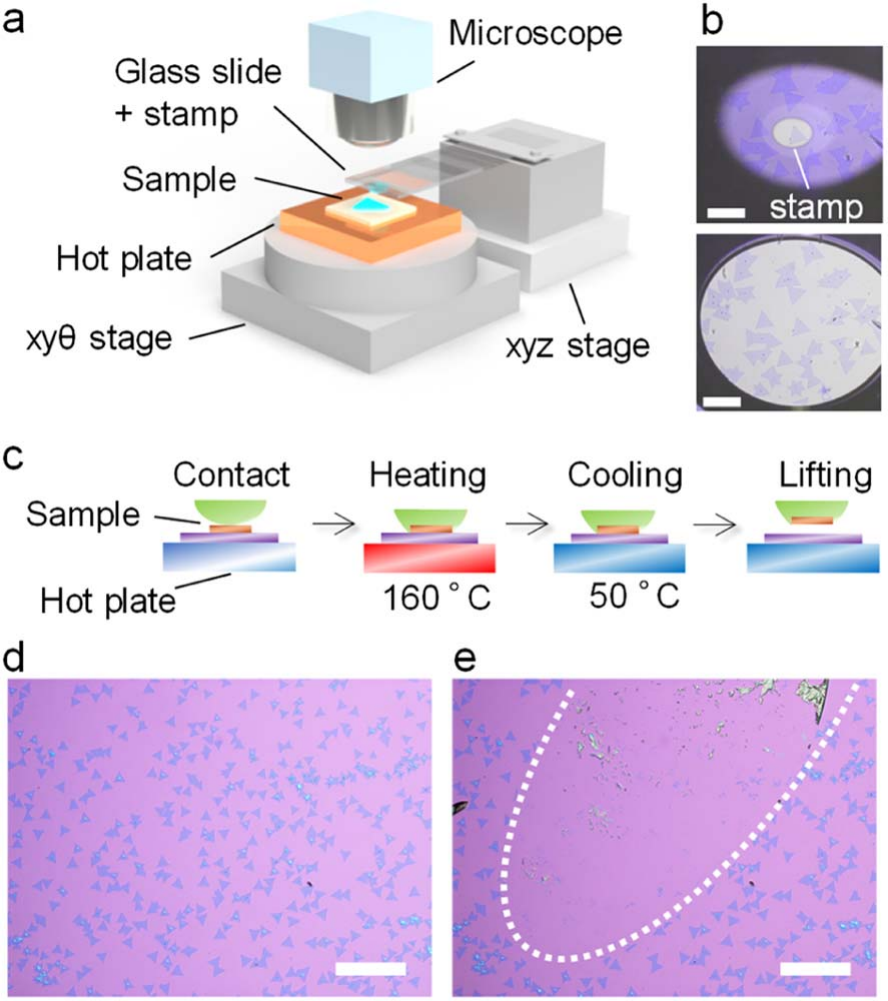
Dry transfer process with the acrylic resin stamp. (a) Schematic of the transfer system used in the present study. (b) Optical images of MoS_2_ grains in contact with the stamp (top) at room temperature and (bottom) during heating. (c) Schematic of the transfer process including (i) the contact of the stamp with the sample, (ii) heating to melt the stamp, (iii) cooling to solidify the stamp, and (iv) lifting the stamp to pick up TMDC monolayers. Optical images of CVD-grown grains of MoS_2_ (d) before and (e) after stamp lifting. The scale bars are 200 μm. White dotted lines indicate the areas in contact with the stamp.

The present dry transfer has been conducted by using an acrylic resin stamp prepared on glass slides, as reported previously.^22^ The stamp was first contacted with the TMDC samples on a hotplate with a motorized xyz stage (Fig. 2a). The sample and stamp were then heated with a hotplate, which softened and spread the acrylic resin around 100 °C (Fig. 2b). To lift the CVD monolayers, the stamp and sample were heated at 160 °C, and then cooled to 50 °C in the present study. After cooling, the stamp was gradually peeled from the substrate (Fig. 2c). Notably, the introduction of melting and solidification processes of the stamp improves the adhesion of the stamp to the monolayer flakes. This improvement enables us to pick up efficiently the single crystals of various TMDC monolayers from the SiO_2_ surface. Fig. 2d and e show the typical optical images of monolayer MoS_2_ grown on a SiO_2_/Si substrate before and after the contact and peeling of stamps, respectively. This process led to the lifting of almost all grains in contact with the stamp. Without cooling, the softened stamp tends to remain on the substrate, and lifting the TMDCs from the substrates is difficult. The lifting yield also depends on other factors such as growth substrates. For example, TMDCs grown on single crystal sapphire substrates can hardly be lifted directly by the stamp probably due to the strong adhesion between TMDCs and single crystal sapphire. Further studies are required to understand the interaction between TMDCs and growth substrates in the future. In the following, we will focus on the PL properties of dry-transferred, CVD-grown TMDCs.

**Fig. 3:**
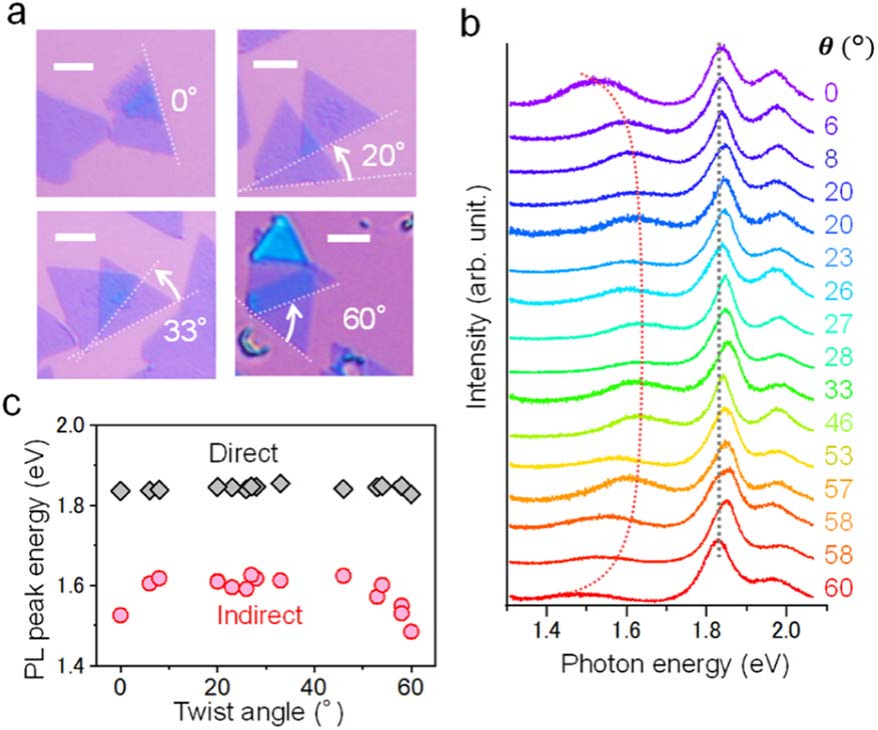
Evaluation of interlayer interaction for twisted bilayer MoS_2_. (a) Optical images of four representative twisted bilayers with different twist angles for CVD-grown MoS_2_. Scale bars are 5 μm. (b) Room-temperature PL spectra and (c) PL peak positions for direct and indirect optical transitions for 16 twisted bilayer MoS_2_ with different twist angles. Black line shows the PL peak of the A exciton of MoS_2_, whereas the red line indicates the PL peaks derived from an indirect gap.

The interlayer interactions of such vdW heterostructures were then investigated by PL spectroscopy for twisted MoS_2_ bilayers. Fig. 3a shows the representative optical images of four twisted MoS_2_ bilayers with different twist angles. The PL spectra of 16 different twisted MoS_2_ bilayers are shown in Fig. 3b. These samples show two prominent PL peaks at 1.8 eV and 1.6 eV, which are due, respectively, to the direct transition associated with the free excitons (A exciton) and the indirect transition.^45^ The A exciton is almost independent of the twist angle, while indirect transitions show a low energy shift at twist angles of 0° and 60° (Fig. 3b and c). These trends are consistent with previous studies and can be explained by the difference in the interlayer distance.^46^ We further investigated the surface quality of these transferred TMDC homobilayers using atomic force microscopy (AFM) as shown in Fig. S1.† The height of the top transferred layer (0.8–0.9 nm) is comparable with that of the bottom layer (0.8–0.9 nm), which indicates a well coupling between the two layers. These results indicate a sufficient interlayer interaction in the present vdW heterostructures obtained by dry transfer of CVD-grown TMDC monolayers.

**Fig. 4:**
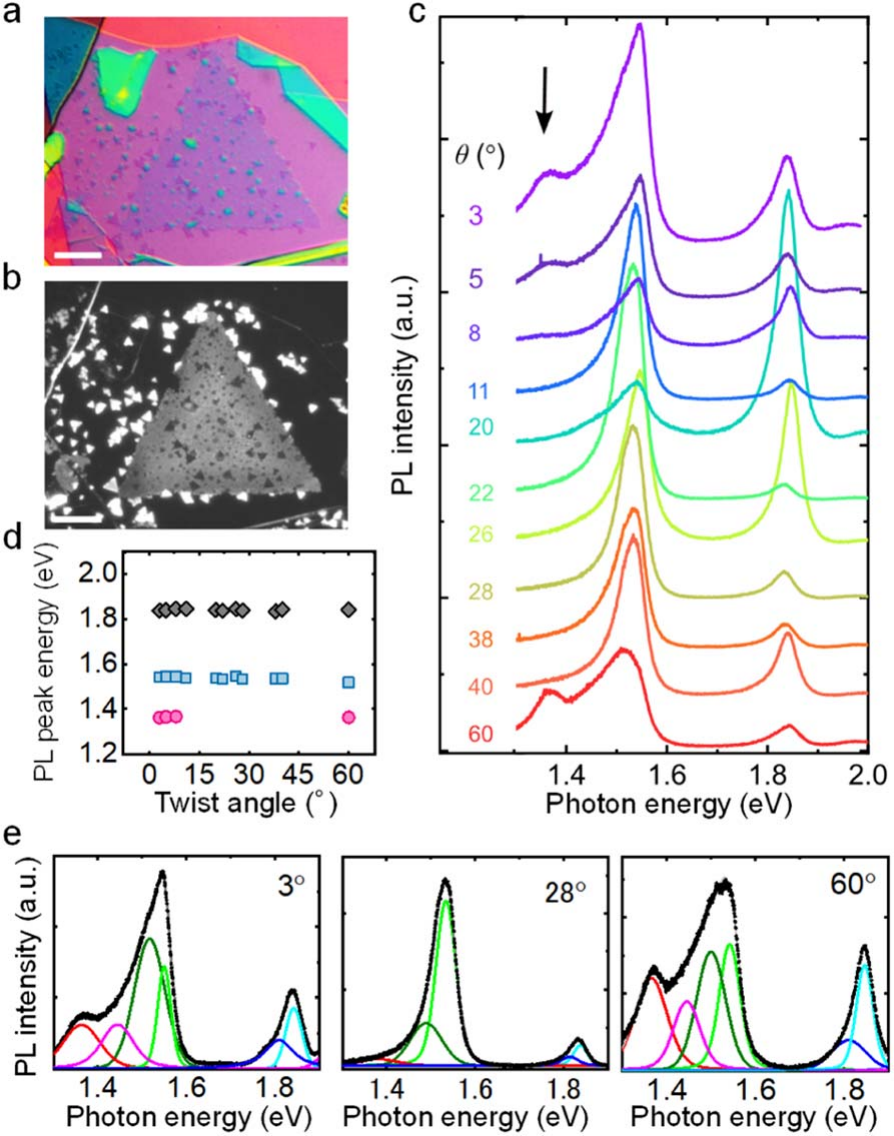
PL properties of MoS_2_/MoSe_2_ heterobilayers with various twist angles. (a) Optical images of hBN encapsulated MoS_2_/MoSe_2_ heterobilayers. (b) PL intensity image (smaller white triangles are 1L MoSe_2_ and larger triangle is 1L MoS_2_, and the dark triangles within MoS_2_ are twisted area). Scale bars are 10 μm. (c) Room-temperature PL spectra of the twisted area with various twist angles. The dashed line indicates the trend of the interlayer exciton peak. (d) PL peak positions of the intralayer exciton from MoS_2_ and MoSe_2_, and the interlayer exciton from the MoSe_2_/MoS_2_ heterobilayer with different twist angles. (e) Fitting results of the PL spectra of the hBN encapsulated MoSe_2_/MoS_2_ twisted bilayers at 3°, 28°, and 60° twist angles.

To improve sample quality, TMDC heterobilayers were encapsulated into hBN by using the present dry transfer process. PL measurements reveal that the hBN encapsulation process releases inhomogeneous lattice strain for monolayer TMDCs grown on the SiO_2_ surface (Fig. S2†). Fig. 4a and b show the optical and PL images of the hBN-encapsulated MoS_2_/MoSe_2_ heterostructure on an SiO_2_/Si substrate, respectively. In Fig. 4b, bright PL can be observed from small triangles of monolayer MoSe_2_ around the larger grain of monolayer MoS_2_. Furthermore, the dark small triangles within the monolayer MoS_2_ single crystal correspond to MoS_2_/MoSe_2_ heterobilayers with various twist angles. The weak PL signal in the stacked area is mainly from the charge and energy transfer induced PL quenching of A excitons of MoS_2_ and MoSe_2_. Fig. 4c shows the PL spectra of these twisted heterobilayers measured at room temperature. Here, clear peaks derived from interlayer excitons were observed at 1.35 eV for the heterobilayers with smaller (or larger) twist angles of 3, 5 and 60°. These peaks can be assigned to interlayer excitons as reported in a previous study.^47^ In contrast, such peaks can be hardly detected for the other intermediate twist angles. This indicates a crystal orientation induced variation in interlayer coupling strength.^48,49^ The PL peak energies are summarized in Fig. 4d. Furthermore, the peak shapes of A excitons at lower (or higher) twist angles become asymmetric and broadened compared with those at intermediate twist angles. The spectral decomposition results show that there is new peak at lower (or higher) twist angles (Fig. 4e), which probably comes from the atomic reconstruction within the moiré unit cell.^14^ Other hBN encapsulated heterobilayers of MoSe_2_/WSe_2_ and MoSe_2_/WS_2_ were also investigated (Fig. S3–S5†). Further investigation including low temperature measurements will be required to fully understand the twist angle dependent excitonic properties of TMDC-based heterobilayers.

**Fig. 5:**
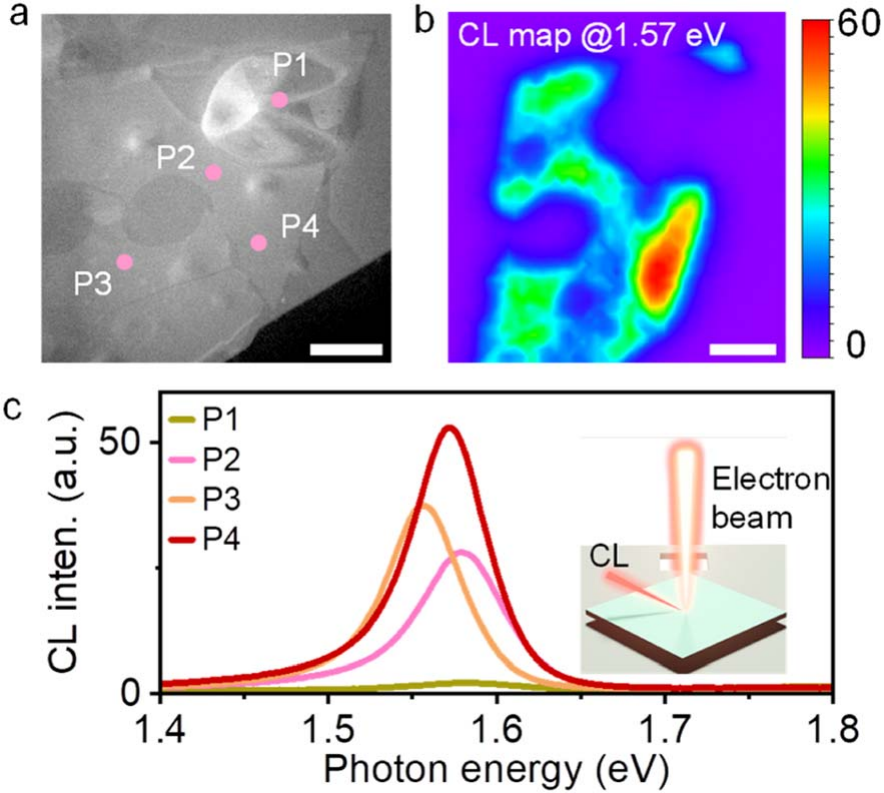
Cathodoluminescence (CL) analysis of hBN-encapsulated monolayer MoSe_2_. (a) STEM image of hBN-encapsulated monolayer MoSe_2_ suspended on a TEM grid. (b) CL map at 1.57 eV of the same area as (a). Scale bars in (a) and (b) are 1 μm. (c) CL spectra recorded at the positions indicated by P1, P2, P3, and P4 in (a). The inset shows a schematic of the CL measurement under electron beam excitation.

Finally, the quality and challenges of dry transfer samples are discussed. In the present samples, the dot-like reduction of PL intensities was frequently observed in the PL intensity map (Fig. S6†). This corresponds to the positions of bubbles formed in the hBN encapsulated monolayer MoS_2_. The peak energies show slight variations within a few tens of meV at different positions. This is derived from the bandgap modulation due to inhomogeneous lattice strain, which was induced by contamination and folding introduced during the present dry transfer process. To obtain local optical properties at higher spatial resolution, a cathodoluminescence (CL) experiment was carried out using a scanning transmission microscope (STEM) equipped with a parabolic mirror and a spectrometer.^50^ In the present system, an electron beam can be focused down to 1 nm scales. Fig. 5a shows the STEM darkfield (DF) image of hBN encapsulated MoSe_2_ on a SiN TEM grid. In the DF image, bubbles and cracks can be clearly identified within the MoSe_2_ grain. The CL map of the same area at 1.57 eV (Fig. 5b) shows weaker signals within the bubbles or cracks compared to the flat areas. As shown in Fig. 5c, the CL spectra at different positions (P1–4) show the variation of the CL intensity and peak energy even for relatively flat regions such as P2, P3, and P4. This indicates the difficulty of obtaining strain-free TMDC samples even after the hBN encapsulation. In future, this issue needs to be addressed through improvements in the transfer and post-transfer processes.

**Fig. 6:**
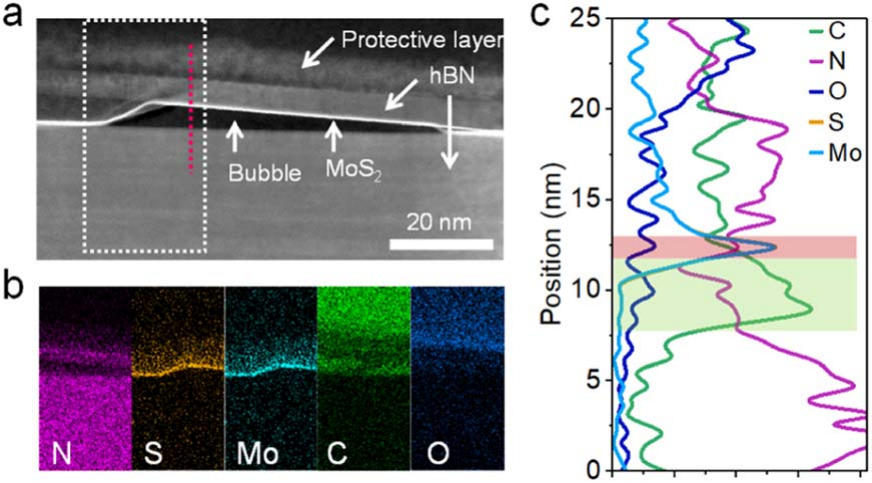
Cross-sectional structural analysis of hBN-encapsulated MoS_2_ stacked in a vacuum. (a) Cross-sectional STEM image with bubbles. (b) EDS mapping of the selected area (dotted rectangle) in (a). (c) Line elemental analysis along the red line in (a). Light red and green regions are MoS_2_ and the bubble area, respectively.

It is noted that the bubble area around P1 shows a quenching of CL, suggesting energy and/or charge transfer to impurities in the bubbles. The CL quenching may also result from the difference in surrounding hBN layers due to the bubble formation. To identify the impurities in bubbles formed in the present process, the cross-sectional structure was observed by STEM. Fig. 6a shows the STEM image of hBN-encapsulated monolayer MoS_2_. This sample was prepared by dry transfer under vacuum (10^−3^ Pa) to reduce the contamination of water and oxygen in air for elemental analysis. It is noted that the bubbles still can be seen as shown in Fig. 6a. Energy dispersive spectroscopy (EDS) mapping shows that carbon is the main element in the bubble area (Fig. 6b and c). In the image, carbon impurities exist between monolayer MoS_2_ and bottom hBN. Considering the hBN exfoliation by adhesive tape, the origin of these carbon impurities is probably from organic materials on the adhesive tape. These impurities may be removed by a post-exfoliation annealing process. Even though further efforts are needed to overcome these disadvantages, it is noteworthy that the present findings provide a clue to improve the sample quality of vdW heterostructures prepared by the dry transfer of CVD-grown TMDCs.

## Conclusions

We have investigated the dry transfer process that employs simple acrylic resin stamps and characterized the optical properties of transferred CVD-grown monolayer TMDCs. The present study demonstrated a simple and rapid way of fabricating a variety of TMDC-based twisted multilayers. To improve the dry transfer process, we introduced the melting and solidification process of the acrylic resin stamp. This improvement allows us to efficiently pick up the single crystals of CVD-grown TMDC monolayers from the SiO_2_ surface. It should be noted that the present transfer is not a fully automated system, although motorized stages were used in our setup. A fully automated system is very powerful to search for monolayer flakes in exfoliated samples, because searching for monolayer flakes with the desired size is the most time-consuming process. Importantly, we can skip the search process by using CVD-grown TMDC samples, where the size and density of the monolayers can be controlled by the growth conditions and the crystal orientation can be easily identified. This allows us to perform high throughput and continuous dry transfer. Because of the simplicity of the process and setup, we believe that this approach is not only scalable but also easy to use for many researchers. PL spectroscopy revealed a sufficient interlayer interaction in the twisted MoS_2_ bilayers obtained by the present dry transfer. Furthermore, the good interlayer coupling was also supported by the observation of room temperature interlayer excitons and their twist angle sensitive properties for hBN encapsulated heterobilayers with various twist angles. The local bandgap modulation and quenching were investigated by CL imaging and spectroscopy. Importantly, nanoscale bandgap variation was observed even for flat regions in the hBN-encapsulated MoS_2_ monolayer. Cross-sectional STEM analysis suggests that the PL/CL quenching is derived from the carbon contamination in bubbles. These findings provide a basis for fabricating high-quality vdW heterostructures. This is also important for investigating the emergent electrical and optical properties of these vdW heterostructures, and for future device applications in electronics and optoelectronics.

## Experimental method

### Sample preparation

TMDC monolayers, including MoS_2_, WS_2_, WSe_2_, and MoSe_2_, were grown on SiO_2_/Si (SiO_2_ thickness: 285 nm) substrates by CVD, as reported previously.^40,51,52^ For large-area WS_2_ growth, the substrate was placed in a quartz tube with WO_3_ powder (300 mg) and sulfur flakes (2 g). After filling the quartz tube with N_2_ gas at atmospheric pressure (and a constant flow rate of 600 sccm), the temperature of the WO_3_ powder was gradually raised to 1050 °C using an electric furnace, to supply W precursors to the downstream substrate. Once the target temperature was reached, the sulfur was heated at 180 °C for 10 min with a second electric furnace. Then, the entire system was immediately cooled using an electric fan. The same growth conditions were employed for MoS_2_, MoSe_2_, and WSe_2_. MoS_2_ was grown with MoO_2_ (100–250 mg) at 850–1000 °C under N_2_ gas (200–300 sccm) for 5–10 min. The growth temperatures were changed to obtain single crystals of different sizes and densities. WSe_2_ was grown with Se beads (2 g) instead of sulfur flakes, and the Se was heated at 385 °C for 2 min under H_2_ (3%)/N_2_ gas at a flow rate of 300 sccm. Similarly, MoSe_2_ was grown with MoO_2_ powder (100 mg) and Se beads (2 g) at 820 °C with a mixed gas of 400 sccm N_2_ and 1.2 sccm H_2_, and the Se was heated at 420 °C for 2 min. Salt-assisted CVD was also employed to prepare monolayer MoS_2_ and WSe_2_ single crystals for hBN encapsulation.^53,54^ Thin flakes of MoS_2_ and hBN were prepared on SiO_2_/Si substrates by mechanical exfoliation of bulk MoS_2_ (SPI supplies) and bulk hBN,^55^ respectively.

### Transfer process

The transfer of TMDC samples was performed through the polymer-assisted lifting and peeling process using acrylic resin stamps of the type reported in a previous study.^22^ First, 1.8 mg of acrylic resin (Elvacite 2552C, Mitsubishi Chemical America) was dissolved in anisole (1.4 mL, Tokyo Chemical Industry Co., Ltd). A droplet of the solution was deposited on a glass slide and then dried on a hotplate at 185 °C for 30 min. This generates a dome-shaped stamp of acrylic resin with a size of 1 mm. The glass slide and the SiO_2_/Si substrate were fixed in a lab-made transfer system with xyz stages, a hot plate, and an optical microscope (Fig. 2a). To lift the TMDC samples from the substrates, the stamp was brought into contact with the samples at room temperature. Each sample was then heated at 160 °C for 10 min on a hotplate. After the heating step, the hotplate was cooled to 50 °C using an electric fan. The stamp was then gradually lifted from the substrate with a motorized stage. To peel the sample from the stamp, the stamp was placed on a target substrate at room temperature and melted at 185 °C. Finally, the acrylic resin on the substrate was washed away with chloroform. For hBN encapsulation, the exfoliated flakes of hBN were lifted by the same process, and used as a stamp to lift the TMDC monolayers. The lifting of the TMDC monolayers is enabled by the strong interlayer vdW interaction of the atomically flat, clean surface of hBN. Finally, hBN/TMDC was placed on another hBN flake for encapsulation.

### Characterization

Optical images were recorded with an optical microscope (Nikon, ECLIPSE-LV100D). PL spectra were recorded by using a micro-spectrometer (Renishaw, inVia) with an excitation laser operating at 532 nm. Low-temperature PL was obtained by using a lab-made optical setup with a cryostat under vacuum conditions (<10^−4^ Pa). A continuous-wave (cw) semiconductor laser operating at 635 nm was used as the excitation source for the PL measurements. The laser was focused using a 50× objective lens. The PL signals were collected using the same objective lens and detected with a cooled charge-coupled device (CCD) through a spectrometer. Cathodoluminescence measurement was carried using a modified STEM (JEM-2100F, JEOL, Japan) at an acceleration voltage of 80 kV. An aluminum parabolic mirror was used to collimate the CL emission from the sample. The signal was finally recorded using a spectrometer with a CCD camera.

## Author contributions

H. N., Y. Ma., W. Z., M. K., K. H., T. O., and T. E. prepared TMDC samples and performed the characterization. T. S. conducted CL measurement. K. W. and T. T. prepared bulk crystals of hBN. H. N. conducted low-temperature PL measurements with K. M. Y. Mi. developed the concept and supervised the project. H. N., Y. Ma, W. Z., H. E. L., Y. N., and Y. Mi. prepared the figures and wrote the paper. All authors discussed the results and commented on the manuscript.

## Conflicts of interest

There are no conflicts to declare.

## Supplementary Material

NA-005-D3NA00371J-s001
