# Supplementary material for: High-throughput dry transfer and excitonic properties of twisted bilayers based on CVD-grown transition metal dichalcogenides
Source: Nanoscale Adv. 2023 Sep 4;5(18):5115–21. doi: 10.1039/d3na00371j (PMC10496764; doi:10.1039/d3na00371j)
Supplement: NA-005-D3NA00371J-s001 [file NA-005-D3NA00371J-s001.pdf]

Electronic Supplementary Material (ESI) for Nanoscale Advances.
This journal is © The Royal Society of Chemistry 2023

Supporting Information for

High-throughput dry transfer and excitonic properties of twisted bilayers based on CVD-grown transition metal dichalcogenides

Hibiki Naito,[a,†] Yasuyuki Makino,[a,†] Wenjin Zhang,[a,†,*] Tomoya Ogawa,[a] Takahiko Endo,[a] Takumi Sannomiya,[b] Masahiko Kaneda,[a] Kazuki Hashimoto,[a] Hong En Lim,[c] Yusuke Nakanishi,[a] Kenji Watanabe,[d] Takashi Taniguchi,[e] Kazunari Matsuda,[f] and Yasumitsu Miyata[a,*]

[a] *Department of Physics, Tokyo Metropolitan University, Hachioji 192-0397, Japan*
[b] *Department of Materials Science and Engineering, Tokyo Institute of Technology, Yokohama 226-8503, Japan*
[c] *Department of Chemistry, Saitama University, Saitama 338-8570, Japan*
[d] *Research Center for Electronic and Optical Materials, NIMS, Tsukuba 305-0044, Japan*
[e] *Research Center for Materials Nanoarchitectonics, NIMS, Tsukuba 305-0044, Japan*
[f] *Institute of Advanced Energy, Kyoto University, Kyoto, 611-0011 Japan*

[†] These authors contributed equally

[*] Authors to whom correspondence should be addressed:
Yasumitsu Miyata, E-mail: ymiyata@tmu.ac.jp;
Wenjin Zhang, E-mail: wjzhang@tmu.ac.jp

S1. AFM characterization of dry-transferred WS_2 homobilayer.

Figure S1a shows the optical and AFM images of dry transferred monolayer WS_2 on WS_2. The AFM image was taken in the dashed area. The AFM image shows a flat surface for the dry transferred WS_2 even after the cleaning. To evaluate the interlayer coupling, the height of the bottom and top transferred WS_2 was measured as shown in Figure S1b,c. A comparable height of the bottom and top transferred WS_2 was observed (0.8–0.9 nm), suggesting a well coupling between the 2D interface.

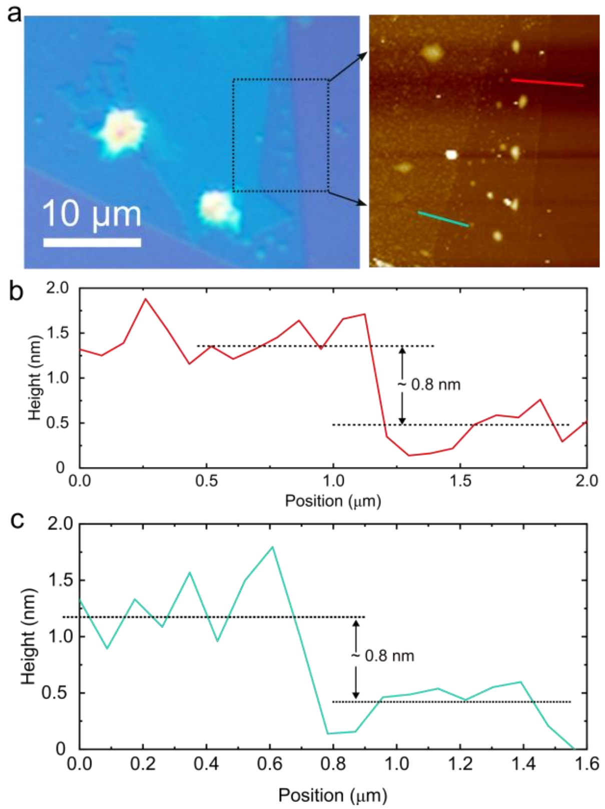

Figure S1. AFM observations of the dry-transferred WS_2 homobilayer. (a) Optical image of homobilayer WS_2 and its AFM topography image in the dashed area. Height profiles of (b) the bottom WS_2 and (c) the top transferred WS_2.

S2. PL spectra of hBN-encapsulated CVD-grown TMDC monolayers.

We have investigated the quality of CVD-grown TMDC monolayers encapsulated in hBN because hBN encapsulation has been used to probe intrinsic transport and optical properties[1-3]. Figure S2a,b shows the optical images and PL intensity maps of CVD-grown monolayer MoS_2 before and after hBN encapsulation. The PL of hBN-encapsulated MoS_2 is 40 times brighter than that of as-grown MoS_2 on the SiO_2/Si substrate. This suggests the suppression of non-radiative relaxation, which could be due to the surface states of SiO_2. In the PL maps, a triangular bright region is observed in the center of the MoS_2 crystal after encapsulation. This reflects the difference in crystallinity. All encapsulated samples, including MoS_2, WS_2, $MoSe_2$, and WSe_2 monolayers, show a high-energy shift and linewidth narrowing of PL peaks compared to the as-grown samples (Figure S2c). This indicates the suppression of inhomogeneous tensile strain as observed for the suspension process[4]. The high quality and the suppression of inhomogeneous broadening were also confirmed for the low-temperature PL spectrum of hBN-encapsulated monolayer $MoSe_2$ (Figure S2d). The PL spectrum measured at 8 K shows two sharp peaks corresponding to the emission from neutral excitons (X^0) and trions (T)[5]. The widths of these peaks are 8.5 meV for X^0 and 7.2 meV for T, which are comparable to the values for the exfoliated flakes encapsulated by hBN[2]. Similar studies on the hBN encapsulation of CVD-grown TMDCs have reported the exciton-exciton annihilation and low-temperature PL for hBN-encapsulated monolayered WS_2[3, 6], and the low-temperature PL and reflectance spectra for WSe_2[7]. Together with these studies, the present results provide a basis for the intrinsic optical properties of CVD-grown TMDCs.

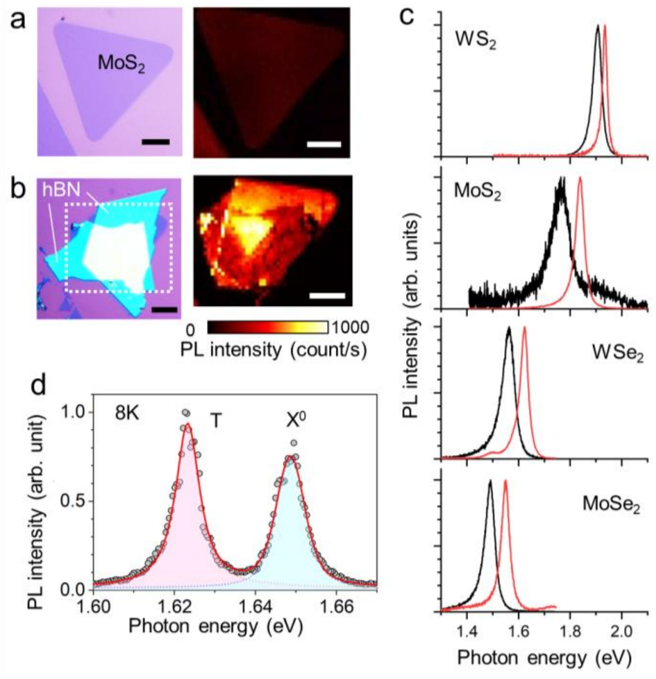

Figure S2. Characterizations of hBN-encapsulated CVD-grown TMDC monolayers. Optical images (left) and PL intensity maps (right) for (a) as-grown monolayer MoS_2 on SiO_2/Si substrate, and (b) hBN-encapsulated MoS_2 on SiO_2/Si substrate. The scale bars are 10 μm. (c) Room-temperature PL spectra of (black) as-grown and (red) hBN-encapsulated monolayer WS_2, MoS_2, WSe_2, and $MoSe_2$. (d) Low-temperature PL spectra of hBN-encapsulated monolayer $MoSe_2$ measured at 8 K. X^0 and T indicate the PL peaks derived from neutral excitons and trions, respectively.

S3. Characterizations of hBN-encapsulated $MoSe_2/WSe_2$ twisted bilayers.

Figure S3a,b shows the optical images and PL intensity maps of hBN-encapsulated $MoSe_2/WSe_2$ twisted bilayers on an SiO_2/Si (sample #1) and on a TEM grid (sample #2). The PL intensity maps at 1.62 eV show the dark triangular regions corresponding to the $MoSe_2/WSe_2$ twisted bilayers. The PL spectra were obtained from the twisted bilayers with different twist angles as shown in Figure S3c. Room temperature interlayer excitons were observed at ~ 1.3 eV for the twist angles of 1 and 60 degrees for sample #1 and 1 and 2 degrees for sample #2. This indicates a well interlayer coupling[8, 9], which is a similar result with the hBN-encapsulated $MoSe_2/MoS_2$ twisted bilayers as described in the main text.

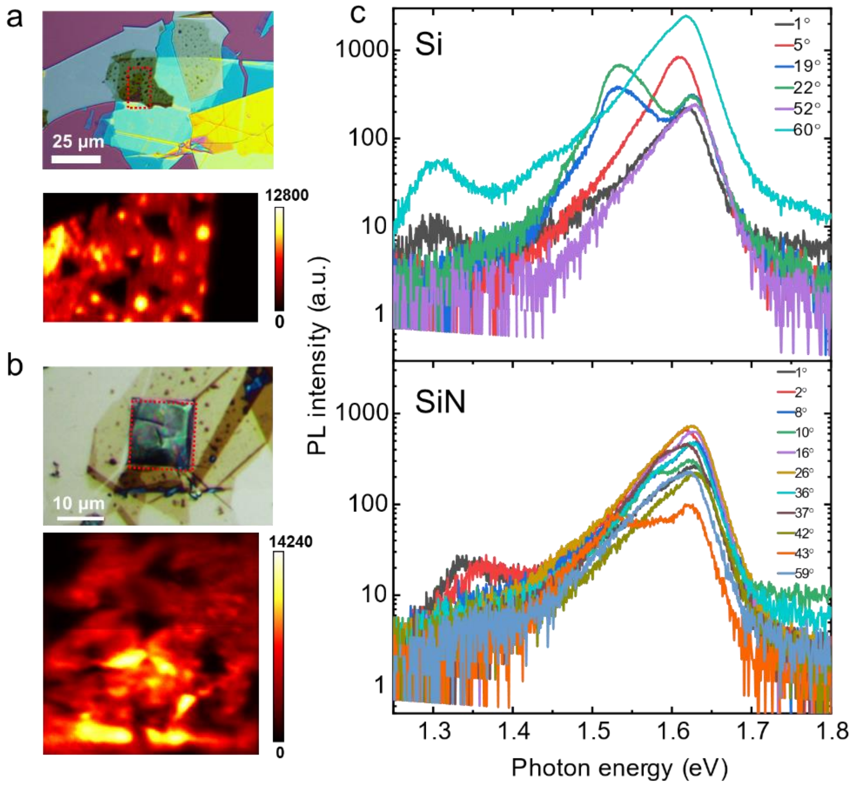

Figure S3. Characterizations of hBN-encapsulated $MoSe_2/WSe_2$ twisted bilayers. Optical micrograph images and PL intensity maps of the red dashed region at 1.62 eV (WSe_2) of the fabricated samples on (a) an SiO_2/Si substrate and (b) a SiN TEM grid. (c) PL spectra of $MoSe_2/WSe_2$ twisted bilayers with different twist angles measured on the SiO_2/Si substrate and the TEM grid.

S4. Characterizations of hBN-encapsulated $MoSe_2/WS_2$ twisted bilayers.

Figure S4a shows the optical micrograph image of hBN encapsulated $MoSe_2/WS_2$ twisted bilayers. The corresponding PL image is shown in Figure S4b, and the green dashed lines indicate the grain edges of monolayer WS_2. Tens of small $MoSe_2/WS_2$ twisted bilayers can be observed within large WS_2 grain. Figure S4c shows a typical broadband PL spectrum from the twisted bilayers. PL peaks of $MoSe_2$ in the twisted bilayers show a red shift compared with that of monolayer $MoSe_2$ as shown in Figure S4d. Note that the twist angle dependence is not as large as reported results[8].

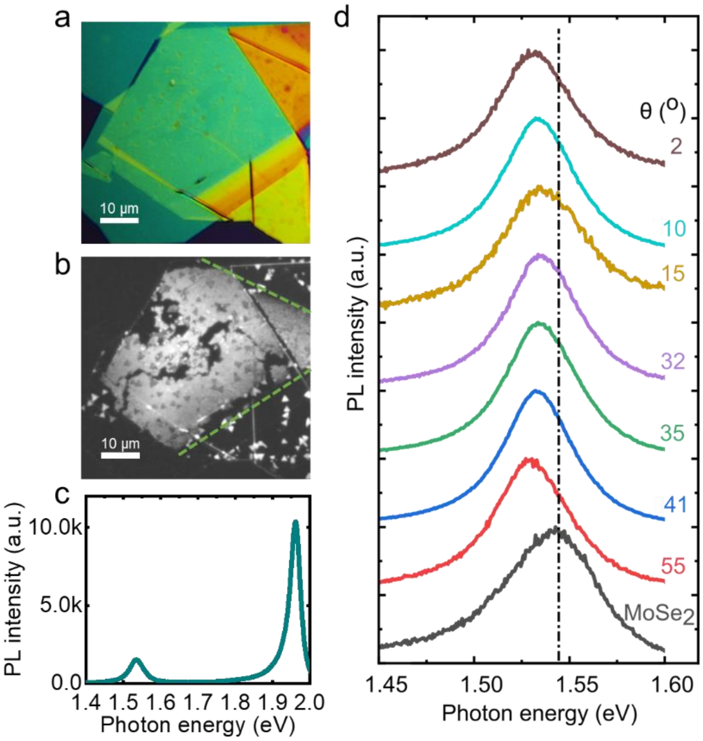

Figure S4. (a) Optical micrograph and (b) PL images of hBN encapsulated $MoSe_2/WS_2$. In (b), the green dashed lines indicate the gain edge of bottom monolayer WS_2. (c) Typical PL spectrum obtained from the twisted bilayer region. (d) Comparison of PL spectra obtained from the twisted region with seven different twist angles and monolayer $MoSe_2$.

S5. Twist-angle dependence of PL peak energies.

For the hBN-encapsulated $MoSe_2/MoS_2$ and $MoSe_2/WSe_2$ samples, PL peaks of interlayer exciton were observed at room temperature. Furthermore, the PL peak energies of interlayer exciton showed a twist angle dependence as shown in Figure S5a. This twist angle dependent interlayer exciton property is likely due to the indirect transition[10], interlayer coupling effect[11], or the effect of atomic reconstruction[12]. For the $MoSe_2/WS_2$, the PL peak energies showed a red shift compared to the A exciton of monolayer $MoSe_2$ (Figure S4d). As shown in Figure S5b, the PL peaks depend on the twist angle (red circles), which results from the hybridization effect of interlayer and intralayer excitons because of the degenerated conduction band edges of $MoSe_2$ and WS_2[13]. For the $MoSe_2/MoS_2$, the A exciton showed a red shift with a twist angle of 60°. A similar red shift was observed in the previous study, where the effect of atomic reconstruction was discussed[12]. Further study is necessary to investigate the origin of each combination in the future, and it is beyond the scope of the current study.

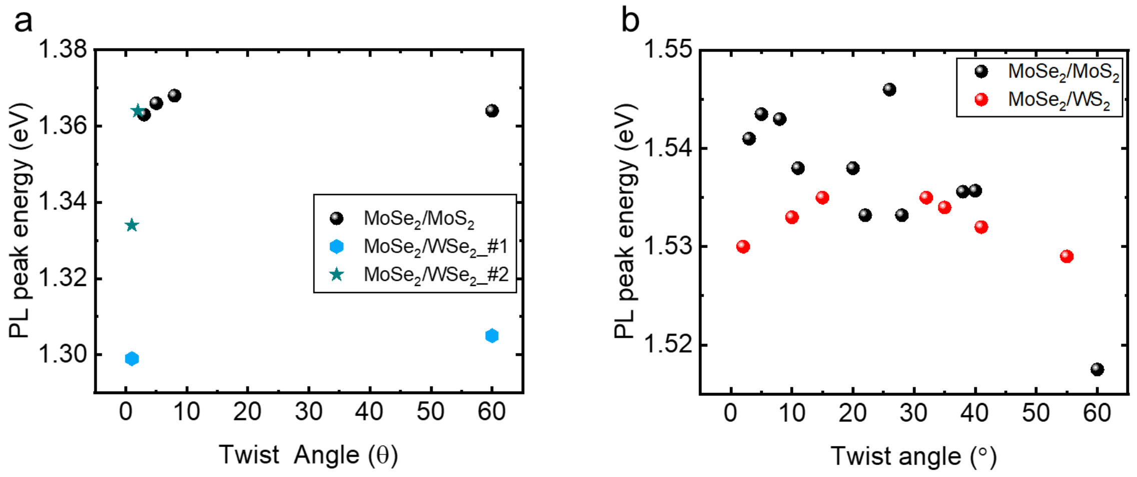

Figure S5. (a) PL peak energies of interlayer exciton of hBN encapsulated $MoSe_2/MoS_2$ and $MoSe_2/WSe_2$ at various twist angles. (b) PL peak energies of A exciton of monolayer $MoSe_2$ of hBN encapsulated $MoSe_2/MoS_2$ and $MoSe_2/WS_2$ at various twist angles.

S6. Characterizations of hBN-encapsulated monolayer MoS_2.

To further characterize the sample quality, hBN-encapsulated monolayer MoS_2 was prepared by the present transfer method as shown in Figure S6a. A PL intensity map at 1.81 eV (under a 532 nm laser excitation) was obtained in the red dashed region as shown in Figure S6b. The PL map shows many quenched regions. The AFM image showed dot-like bubbles at the same locations with PL quenched regions (Figure S6c). In Figure S6d, the PL spectra measured at the bubbles show variations in PL intensity and peak energy. This is likely due to the bandgap modulation by lattice strain[14] and the impurity-assisted nonradiative relaxation around the bubbles.

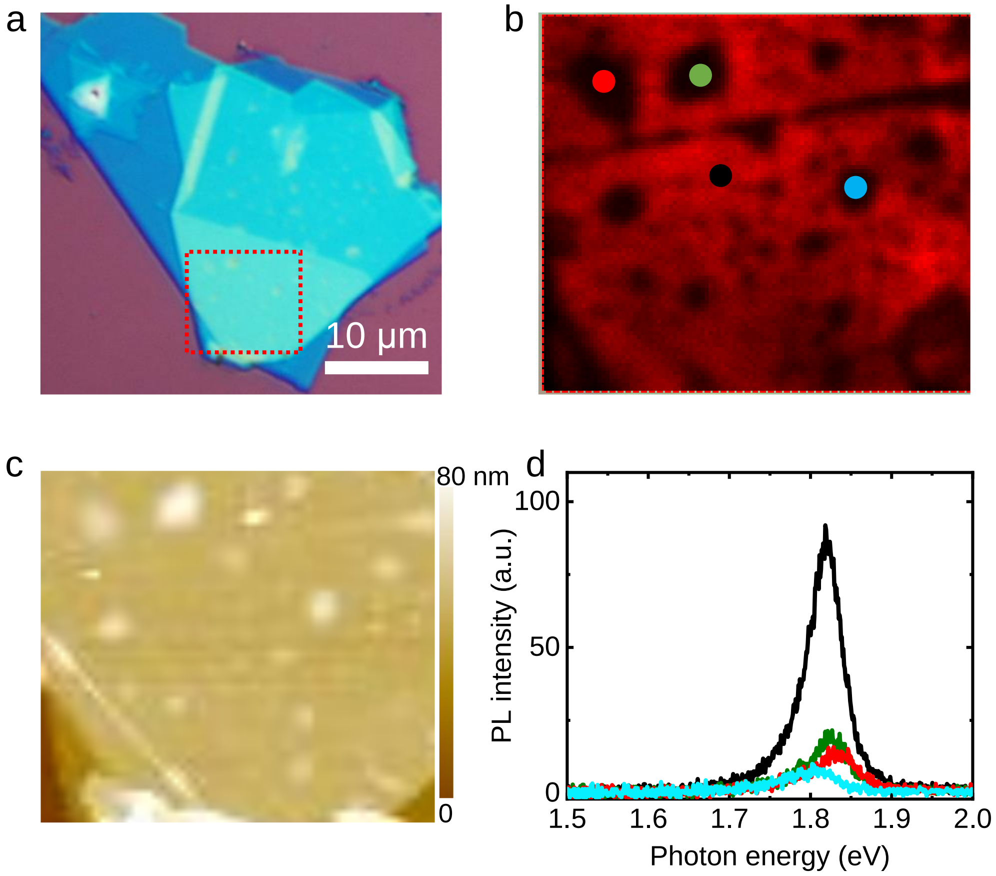

Figure S6. (a) Optical micrograph image of hBN-encapsulated monolayer MoS_2. (b) PL intensity map (@1.81 eV) and (c) AFM image of the red dashed area in (a). (d) Typical PL spectra taken from the marked area in (b).

References:

1. L. Wang, I. Meric, P. Y. Huang, Q. Gao, Y. Gao, H. Tran, T. Taniguchi, K. Watanabe, L. M. Campos, D. A. Muller, J. Guo, P. Kim, J. Hone, K. L. Shepard and C. R. Dean, *Science*, 2013, **342**, 614-617.
2. F. Cadiz, E. Courtade, C. Robert, G. Wang, Y. Shen, H. Cai, T. Taniguchi, K. Watanabe, H. Carrere, D. Lagarde, M. Manca, T. Amand, P. Renucci, S. Tongay, X. Marie and B. Urbaszek, *Phys. Rev. X*, 2017, **7**, 021026.
3. Y. Hoshi, T. Kuroda, M. Okada, R. Moriya, S. Masubuchi, K. Watanabe, T. Taniguchi, R. Kitaura and T. Machida, *Phys. Rev. B*, 2017, **95**, 241403.
4. K. Kojima, H. E. Lim, Z. Liu, W. Zhang, T. Saito, Y. Nakanishi, T. Endo, Y. Kobayashi, K. Watanabe, T. Taniguchi, K. Matsuda, Y. Maniwa, Y. Miyauchi and Y. Miyata, *Nanoscale*, 2019, **11**, 12798-12803.
5. J. S. Ross, S. Wu, H. Yu, N. J. Ghimire, A. M. Jones, G. Aivazian, J. Yan, D. G. Mandrus, D. Xiao, W. Yao and X. Xu, *Nat. Commun.*, 2013, **4**, 1474.
6. Y. Hoshi, M. Okada, R. Moriya, S. Masubuchi, K. Watanabe, T. Taniguchi, R. Kitaura and T. Machida, *Phys. Rev. Mater.*, 2018, **2**, 064003.
7. A. J. Mannix, A. Ye, S. H. Sung, A. Ray, F. Mujid, C. Park, M. Lee, J.-H. Kang, R. Shreiner, A. A. High, D. A. Muller, R. Hovden and J. Park, *Nat. Nanotechnol.*, 2022, **17**, 361-366.
8. K. Wang, B. Huang, M. Tian, F. Ceballos, M. W. Lin, M. Mahjouri-Samani, A. Boulesbaa, A. A. Puretzky, C. M. Rouleau, M. Yoon, H. Zhao, K. Xiao, G. Duscher and D. B. Geohegan, *ACS Nano*, 2016, **10**, 6612-6622.
9. M.-H. Chiu, M.-Y. Li, W. Zhang, W.-T. Hsu, W.-H. Chang, M. Terrones, H. Terrones and L.-J. Li, *ACS Nano*, 2014, **8**, 9649-9656.
10. J. Kunstmann, F. Mooshammer, P. Nagler, A. Chaves, F. Stein, N. Paradiso, G. Plechinger, C. Strunk, C. Schüller, G. Seifert, D. R. Reichman and T. Korn, *Nat. Phys.*, 2018, **14**, 801-805.
11. P. K. Nayak, Y. Horbatenko, S. Ahn, G. Kim, J.-U. Lee, K. Y. Ma, A.-R. Jang, H. Lim, D. Kim, S. Ryu, H. Cheong, N. Park and H. S. Shin, *ACS Nano*, 2017, **11**, 4041-4050.
12. B.-H. Lin, Y.-C. Chao, I. T. Hsieh, C.-P. Chuu, C.-J. Lee, F.-H. Chu, L.-S. Lu, W.-T. Hsu, C.-W. Pao, C.-K. Shih, J.-J. Su and W.-H. Chang, *Nano Letters*, 2023, **23**, 1306-1312.
13. E. M. Alexeev, D. A. Ruiz-Tijerina, M. Danovich, M. J. Hamer, D. J. Terry, P. K. Nayak, S. Ahn, S. Pak, J. Lee, J. I. Sohn, M. R. Molas, M. Koperski, K. Watanabe, T. Taniguchi, K. S. Novoselov, R. V. Gorbachev, H. S. Shin, V. I. Fal'ko and A. I. Tartakovskii, *Nature*, 2019, **567**, 81-86.
14. C. R. Zhu, G. Wang, B. L. Liu, X. Marie, X. F. Qiao, X. Zhang, X. X. Wu, H. Fan, P. H. Tan, T. Amand and B. Urbaszek, *Phys. Rev. B*, 2013, **88**, 121301.
